# Molecular Dynamic Investigations on the Adhesion Behaviors of Asphalt Mastic–Aggregate Interface

**DOI:** 10.3390/ma13225061

**Published:** 2020-11-10

**Authors:** Wenyi Xu, Xin Qiu, Shanglin Xiao, Ganghua Hu, Feng Wang, Jie Yuan

**Affiliations:** 1 College of Engineering, Zhejiang Normal University, Jinhua 321004, China; wenyixu@zjnu.edu.cn (W.X.); sl-xiao@zjnu.cn (S.X.); zjnuhugh@outlook.com (G.H.); 2 Key Laboratory of Urban Rail Transit Intelligent Operation and Maintenance Technology & Equipment of Zhejiang Province, Zhejiang Normal University, Jinhua 321004, China; 3 Ingram School of Engineering, Texas State University, San Marcos, TX 78666, USA; f_w34@txstate.edu; 4 Key Laboratory of Road and Traffic Engineering of the Ministry of Education, Tongji University, Shanghai 201804, China; yuanjie@tongji.edu.cn

**Keywords:** asphalt–aggregate interface, asphalt mastic, molecular dynamic, molecular arrangement, nanostructure, moisture damage

## Abstract

The asphalt mastic–aggregate interface plays an essential role in determining the service performance of asphalt mixtures. The objective of this paper was to investigate the adhesion behaviors and mechanism between asphalt mastic and aggregate based on molecular dynamic (MD) simulations. First, the asphalt mastic model considering the actual mass ratio of filler to asphalt (F/A) condition was established and validated in terms of thermodynamic properties. Second, the molecular arrangement characteristics of polar components on the aggregate substrate were analyzed by radial distribution function (RDF), relative concentration (RC), and mean square displacement (MSD). Third, the interfacial adhesion ability between asphalt and aggregate was quantitively evaluated based on the work of adhesion. Finally, the coupling effect of moisture and temperature on interfacial adhesion behaviors was investigated to explore the adhesion failure characteristics of the asphalt–aggregate interface. The results demonstrate that the thermodynamic properties could be employed to validate the reliability of the asphalt mastic model. The self-aggregation degree of polar components in base asphalt could be significantly increased with the addition of silica particles, exhibiting a change of configuration from “parallel arrangement” into “stack distribution” due to the high polarity of silica particles. The polar components in asphalt mastic exhibit a more uniform distribution state and lower mobility capability than base asphalt owing to the adsorption effect of silica particles. Silica particles with amounts of residual charges could significantly increase the electrostatic energy of the asphalt mastic–aggregate interface, contributing to an improvement of the adhesion between asphalt mastic and aggregate. The increase of temperature enhances the work of adhesion of the asphalt mastic–aggregate interface, which is opposite to that of the base asphalt–aggregate interface. The asphalt mastic exhibits a greater sensitivity to interfacial moisture damage than base asphalt. The findings would provide insights into a better understanding on the micro adhesion mechanism of the asphalt mastic–aggregate interface.

## 1. Introduction

During the long-term service period, asphalt mixtures are extremely prone to damage due to environmental factors and vehicle loads [1,2,3]. Previous studies have proven that the asphalt–aggregate interface is the most fragile position in asphalt mixtures, which has a significant influence on the overall performance of asphalt mixtures [4,5]. For example, the presence of moisture, aging, and temperature, among others, would dramatically reduce the asphalt–aggregate interfacial bonding strength, leading to the deterioration of the service performance of asphalt mixtures [6,7,8,9]. Therefore, deeply understanding adhesion behaviors and improving adhesion ability of the asphalt–aggregate interface are of great significance for reducing the occurrence of various distresses and prolonging the service life of asphalt pavement.

To date, some fundamental theories have contributed to explaining the adhesion mechanism between the asphalt and aggregate such as mechanical theory [10], chemical theory [11], weak boundary theory [12,13], and thermodynamic theory [14]. The mechanical theory describes adhesion as the mechanical interlock between the asphalt binder and aggregate surface. It is generally accepted that aggregates with porous and slightly rough surfaces could enhance the mechanical interlocking effect and promote a better adhesion with the asphalt binder. The chemical theory indicates that the chemical reaction between acid functional groups in asphalt and alkaline active components on the aggregate surface makes a great contribution to the interfacial bonding ability. The weak boundary theory demonstrates that the adhesive failure of the interface is attributed to the presence of a low adhesion strength region owing to the selective adsorption effect. The thermodynamic theory suggests that the process of energy exchange and eventual equilibrium between asphalt and aggregate is the main reason for the formation of interfacial bonding strength. As for evaluation methods, the adhesion properties of the asphalt–aggregate interface could be quantitively evaluated by different methods, especially the boiling method, which has been commonly utilized as a standard method [15]. Besides, extensive exploratory experimental approaches such as dynamic shear rheometer (DSR) [16], surface free energy method [17,18,19,20], and atomic force microscopy (AFM) [21,22,23,24,25], have also been carried out to quantify the asphalt–aggregate interfacial adhesion behaviors from a multi-scale perspective. However, it is worth mentioning that these theories and techniques still have limitations in explaining the micro adhesion behaviors between asphalt and aggregate from the molecular scale. Therefore, it is urgently needed to take an in-depth investigation on the interfacial adhesion mechanism from the point of view of the characteristics of interfacial nanostructures and molecular arrangement under different circumstances.

Molecular dynamic (MD) simulation is a practical approach to investigate the mechanical, thermodynamic, and structural properties of materials. Especially for asphalt materials, significant efforts have been made to investigate the asphalt–aggregate interfacial adhesion behaviors. Generally, the quantitative evaluation of aggregation and distribution characteristics of asphalt components on the aggregate surface is specifically essential for the adhesion behaviors of the asphalt–aggregate interface. Luo et al. investigated the temperature effect on the diffusion of asphalt components on the mineral aggregate surface with mean square displacement (MSD) and diffusion coefficient. The results demonstrated that the interaction between asphalt components and Al_2_O_3_ aggregate could make the diffusion of asphalt components independent of temperature [26]. Huang et al. studied the diffusion characteristics of asphalt components on mineral surfaces. The results presented that polar fractions, including asphaltene and resin, might cause the physical adsorption of asphalt on the aggregate surface, which is the intrinsic reason for the interfacial adhesion behaviors [27]. Guo et al. analyzed the relative concentration (RC) of asphalt components on the aggregate surface, demonstrating that asphalt components were mainly distributed along with the direction perpendicular to the surface of minerals [28]. Dong et al. characterized the nanostructure of the asphalt–aggregate interface and quantified the aggregation and distribution characteristics with radial distribution function (RDF) and RC, respectively. The results indicated that the aggregation of polar components would provide stronger adhesion ability to resist interfacial damage [29]. Xu et al. applied the stress-separation responses to quantitatively evaluate the bonding strength of the asphalt–aggregate interface, and concluded that the large air voids formed in the bulk asphalt would result in the adhesive failure [30].

Meanwhile, many studies discussed the affecting factors that influence the interfacial adhesion behaviors based on MD simulation. Wang et al. studied the interface stress of the asphalt–quartz aggregate system by taking the moisture and the temperature into account. The results showed that the interface stress declined with the increase of the moisture content or the temperature [31]. Xu et al. studied the adhesion behaviors of aged asphalt at dry and wet conditions and found that moisture would significantly deteriorate the adhesion strength between aged asphalt and aggregate [32]. Sun et al. investigated the effect of aging and moisture on the nanostructure and adhesive energy of the asphalt–aggregate interface and found that aging and moisture could make asphaltene clusters closer. Meanwhile, the moisture would separate the asphaltene and resin away from the mineral surface, leading to the decrease of the interfacial adhesive energy [33]. Gao et al. evaluated the effect of moisture on the bonding and debonding behaviors between asphalt and four types of minerals, including quartz, calcite, albite, and microcline. The results demonstrated that the resistance to moisture damage of the asphalt–aggregate interface was directly dependent on the chemistry of the mineral surface, and the alkali minerals would exhibit stronger non-bonding interaction energy under wet conditions [34]. Liu et al. explored the interaction mechanism between asphalt and steel slag, indicating that the steel slag would provide a stronger electrostatic interaction with asphalt because a large amount of Ca^2+^ is contained in steel slag, which could strengthen the interfacial adhesion ability [35]. Moreover, the process of adding and mixing of material ingredients should be given full consideration in establishing the simulation models, which would influence the adhesion properties of engineering materials [36]. As mentioned above, numerous studies have evaluated the asphalt–aggregate interfacial bonding strength and explored the influential factors of interfacial adhesion behaviors based on the molecular scale, which give support for further research on the adhesion behaviors of the asphalt–aggregate interface.

Actually, an asphalt mixture is a kind of dispersion system with a multi-level spatial network structure, which consists of a coarse dispersion system (asphalt mixture), a subdivided dispersion system (asphalt mortar), and a differential dispersion system (asphalt mastic) [37]. In an asphalt mixture, coarse aggregate as the dispersed phase is distributed in asphalt mortar. As for asphalt mortar, fine aggregate as the dispersed phase is scattered in asphalt mastic, which consists of mineral filler and asphalt binder. Asphalt mastic plays an important role in bonding aggregates and filling voids for asphalt mixtures, which would exert great influence on the pavement performance of asphalt mixtures [38,39,40]. Currently, the technology of MD simulations has been employed to investigate the physical and rheological properties of asphalt mastic. Zhu et al. generated the asphalt mastic molecular model by adding silica molecules for the first time and explored the thermodynamic and mechanical behaviors of asphalt mastic [41]. Li et al. established an asphalt mastic model with a low silica particle content and found that the selective adsorption between silica particles and polar components in asphalt is the potential reason for the physicochemical interaction [42]. Additionally, the exploration of the thermodynamic and mechanical behaviors of nanoparticle-modified asphalt had been conducted through MD simulations, such as adding nano hydrated lime [43], nano-ZnO [44], graphite nanoplatelets [45,46], and carbon-nanotubes [47]. It is worth mentioning that Long et al. analyzed the interfacial adhesion properties of nano-silica modified asphalt mastic through MD simulation. The results indicated that the nano-silica particles as mineral filler would slightly increase the interfacial adhesion between nano-silica modified asphalt and aggregate [48]. The studies would be helpful to establish molecular models of asphalt mastic and to understand its mechanical behaviors from the molecular scale.

In summary, previous studies have enhanced our understanding on the adhesion mechanism and behaviors of the asphalt–aggregate interface through MD simulations. However, to date, little attention has been given to establish molecular models of asphalt mastic in an actual filler to asphalt ratio (F/A) condition and to explore the corresponding thermodynamic behaviors of asphalt mastic. Furthermore, there is a lack of studies on the adhesion mechanism and behaviors of the asphalt mastic–aggregate interface from the perspective of interfacial nanostructure and molecular arrangement. Therefore, the objectives of this study were to explore the aggregation and distribution behaviors of asphalt mastic on the aggregate surface, and to investigate the interfacial adhesion mechanism between asphalt mastic and aggregate with the coupling effect of moisture and temperature based on the molecular arrangement characteristics of asphalt mastic on aggregate surface. First, the asphalt mastic model under the actual F/A condition was established and validated by analyzing the difference of thermodynamic properties between asphalt mastic and base asphalt. Second, an asphalt mastic–aggregate interface model was constructed to study the aggregation and distribution behaviors of polar components on the aggregate substrate from a perspective of interfacial nanostructure and molecular arrangement based on radial distribution function (RDF), relative concentration (RC), and mean square displacement (MSD). Finally, the adhesion strength of the asphalt mastic–aggregate interface was quantitively evaluated considering the coupled effect of moisture and temperature on interfacial adhesion behaviors. The study would hopefully give an insight into understanding the adhesion mechanism between asphalt mastic and aggregate and provide a more reasonable and reliable method for evaluating the interfacial damage resistance of the asphalt mixture.

## 2. Molecular Model Generation

### 2.1. Asphalt Binder

**Figure 1:**
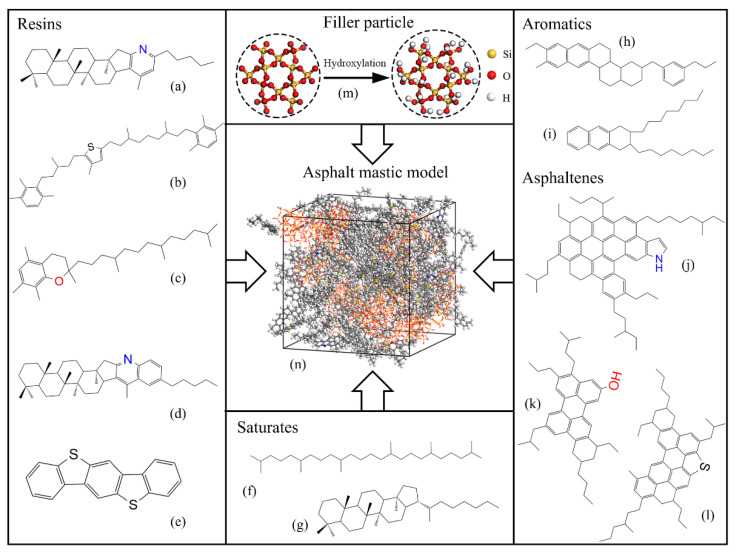
Molecular structures of asphalt mastic. Resins: (**a**) pyridinohopane, (**b**) thio-isorenieratane, (**c**) trimethylbenzene-oxane, (**d**) quinolinohopane, and (**e**) benzobisbenzothiophene; saturates: (**f**) squalene and (**g**) hopane; aromatics: (**h**) perhydrophenanthrene-naphthalene (PHPN) and (**i**) dioctyl-cyclohexane-naphthalene (DOCHN); asphaltenes: (**j**) pyrrole, (**k**) phenol, and (**l**) thiophene; (**m**) mineral filler particle model; (**n**) asphalt mastic model.

Asphalt binder is a complex chemical mixture with millions of high molecular hydrocarbons and nonmetallic derivatives molecules containing sulphur, nitrogen, and oxygen atoms, which makes it difficult to precisely characterize all molecular components and accurately evaluate their properties [49,50]. Fortunately, according to the Corbett separation method [51], asphalt binder could be divided into SARA fractions, such as saturate (S), aromatic (A), resin (R), and asphaltene (A), which provides an effective method for investigating the typical structure of asphalt binder. To understand the physical-mechanical and rheological properties of asphalt binder, the 12-component molecular model of the SHRP AAA-1 asphalt based on SARA fractions, as developed by Li and Greenfield [52], was employed in the study. The molecular characteristics of asphalt components are presented in Table 1 and the molecular structures are illustrated in Figure 1a–l.

### 2.2. Mineral Filler

Mineral filler is ground from mineral aggregates with complex compositions. It is a great challenge to entirely characterize all components in MD simulations. As the silica is the main component of most mineral fillers, the silica model was selected to simplify the structural model of mineral filler in the study. Firstly, a unit crystalline silica was chosen from the structures database in Materials Studio with the lattice parameters of a = b = 4.909 Å, c = 5.402 Å, α = β = 90°, and γ = 120°. Then, the alpha-quartz supercell crystal structure was generated with the supercell range parameters of A = 2, B = 1, and C = 2. Finally, the silica nanocluster with a radius of 5 Å was established, in which the atoms outside the radius were discarded [53]. Besides, the -H and -OH was added at the surface of oxygen atoms and silica atoms in order to keep the silica nanocluster electrically neutral, respectively [54]. As shown in Figure 1m, the silica nanocluster is composed of 16 Si atoms, 50 O atoms, and 24 H atoms.

### 2.3. Asphalt Mastic

During the asphalt mastic model construction, the asphalt molecules and silica particles were randomly distributed in an empty cubic box with an initial density of 0.6 g/cm^3^ to ensure that the distribution of molecules is uniform and the molecular chains are distorted in the model [55]. According to the Superpave volumetric mix design method [56], the mass ratio of filler to asphalt (F/A) was recommended at 0.6–1.2 (the mass fraction is 37.5–54.5%), which was less than the critical volume fraction of 40% to avoid particle structuralization [57,58]. According to [39,59], the F/A of 1.2 was utilized in this study because the asphalt mastic with 1.2 F/A would display better interaction behaviors with aggregates. After the asphalt mastic models were established, the geometry optimization process with the Smart algorithm was first carried out with 5000 iterations to minimize the system energy and optimize the structure. Then, the annealing procedure was conducted for five annealing cycles, wherein the temperature was raised from 273.15 K to 500.15 K for 1 ns and then cooled to 298.15 K for 1 ns to remove unstable configurations. After that, the models were subjected to dynamic equilibration under the NPT ensemble (N: constant particle number, P: constant pressure, T: constant temperature) at 1 atm for 500 ps at 298.15 K to achieve an ideal structure. Finally, an NVT ensemble (V: constant volume) was further conducted for 500 ps at 298.15 K for obtaining the final equilibrium structure with the stable volume and energy. The asphalt mastic model is shown in Figure 1n. The equilibrated sizes of base asphalt and asphalt mastic are 37.8 Å × 37.8 Å × 37.8 Å and 44.4 Å × 44.4 Å × 44.4 Å, respectively.

### 2.4. Asphalt Mastic–Aggregate System

**Figure 2:**
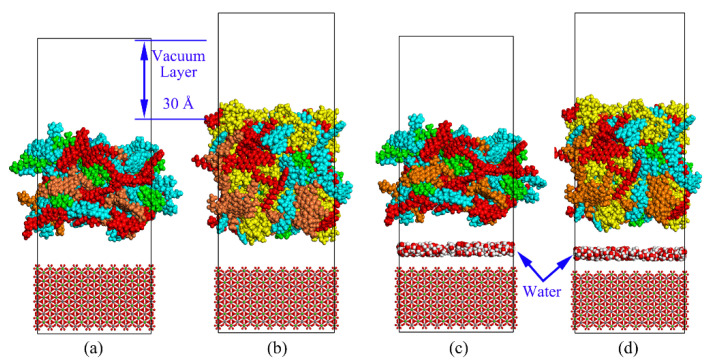
Interface models under dry and wet conditions. (**a**) Base asphalt–aggregate interface model; (**b**) asphalt mastic–aggregate interface model; (**c**) base asphalt–water–aggregate interface model; (**d**) asphalt mastic–water–aggregate interface model (orange: asphaltene; blue: resin; red: aromatic; green: saturate; yellow: silica particles).

Calcite (CaCO_3_) was selected as a representative alkaline mineral aggregate in MD simulations. To construct the aggregate substrate, the unit cell of the calcite model was first cleaved along the (1 0 0) direction to form a corresponding surface with a 5 Å fractional thickness. Then, the crystal surfaces were extended to generate a supercell model by repeating the unit cell in the x-direction and the y-direction after the geometry transformation. Finally, the vacuum slab was included to create the aggregate substrate. After that, the asphalt mastic–aggregate interface model was established by attaching the asphalt mastic layer on the constrained aggregate substrate. A vacuum layer of 30 Å was also added on the top of the asphalt mastic layer to eliminate the periodic boundary condition in the z-direction. Considering that the adhesion failure is the dominant reason for the moisture damage in asphalt mixture [60,61], a thin layer containing 200 water molecules was added into the asphalt mastic–aggregate interface to investigate the effect of moisture on the asphalt mastic–aggregate interfacial bonding behaviors based on the confined layer model. The same process was conducted to generate the base asphalt–aggregate interface model. The interface models for base asphalt–aggregate and asphalt mastic–aggregate under dry and wet conditions are illustrated in Figure 2. After establishing the interface models, the geometry optimization process was initially carried out with 5000 iterations. Then, the annealing processes with the temperature ramping from 273.15 K to 500.15 K for 1 ns and back to room temperature (298.15 K) and the critical high temperature of in-service pavement (343.15 K) for 1 ns, respectively, were performed. Finally, the dynamic equilibration procedures run of 500 ps at 298.15 K and 343.15 K with the NVT ensemble were conducted on all interface models for further analysis. The equilibrated sizes of the base asphalt–aggregate interface and asphalt mastic–aggregate interface are 37.8 Å × 37.8 Å × 103.3 Å and 44.4 Å × 44.4 Å × 117.6 Å, respectively.

### 2.5. Force Field and Simulation Details

The force field is the basis of the molecular dynamic simulations, which could be utilized to accurately describe the molecular trajectory on the potential energy surface in the system. In other words, the force field is closely related to the interactions between atoms, including molecular interactions, intermolecular potentials, and hydrogen bond interactions, among others. An appropriate force field could accurately reflect the essential properties of material systems [34]. The Condensed-Phase Optimized Molecular Potentials for Atomistic Simulation Studies (COMPASS) force field has been validated in simulating asphalt properties and investigating the molecular interactions in the mixed system [62,63]. In this study, the COMPASS force field was applied to evaluate the bond and non-bond interactions of all atoms as well as the charges of all atoms in the system. The processes of molecular dynamic simulations were performed using commercially available simulation software, Materials Studio. During the MD simulations, the Nose–Hoover thermostat and Berendsen barostat were employed to provide a constant temperature and pressure for the model systems. The electrostatic force was calculated based on the Ewald simulation method with a 6 Å repulsive cutoff distance and the Van der Waals force was determined through the atom-based simulation method with a distance of 15.5 Å. Meanwhile, all the simulations were performed with a time step of 1 fs and the dynamic trajectory data were outputted every 5000 steps.

## 3. Results and Discussion

### 3.1. Thermodynamic Properties of Asphalt

The thermodynamic properties including density, glass-transition temperature (*T_g_*), and cohesive energy density (CED) of two kinds of asphalt material models (i.e., base asphalt and asphalt mastic) were discussed to validate whether the molecular simulation method with the COMPASS force field parameters is suitable for describing the characteristics of base asphalt and asphalt mastic.

**Figure 3:**
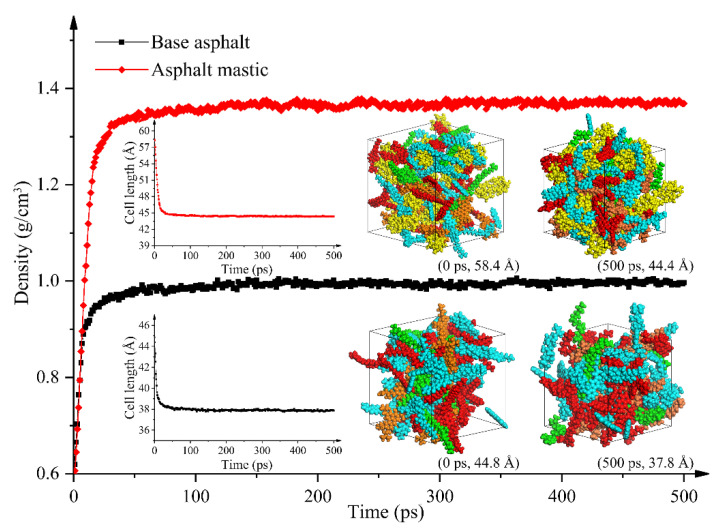
Density results for base asphalt and asphalt mastic.

The density as a crucial thermodynamic property of asphalt is commonly employed to verify the reliability of molecular models in MD simulations. The densities of base asphalt and asphalt mastic were calculated during the NPT ensemble for 500 ps at 1 atm and their values gradually stabilize as the optimization process approaches 500 ps, as shown in Figure 3, at which the densities of base asphalt and asphalt mastic tend to 0.998 g/cm^3^ and 1.371 g/cm^3^, respectively. The calculated density of base asphalt is close to the results, as shown in Table 2. With further analysis, the higher hydrogen, more carbon, but less sulfur and a lack of heteroatoms utilized in asphalt binder models would be the main reason for the simulated results being lower than that of the true density [64]. Besides, as the optimization time increases, the cell length of the models also tends to a stable value. The cell length values of base asphalt and asphalt mastic models range from 44.8 Å to 37.8 Å and from 58.4 Å to 44.4 Å, respectively. Obviously, the addition of filler particles would significantly increase the density of base asphalt and enlarge the model size of base asphalt.

**Figure 4:**
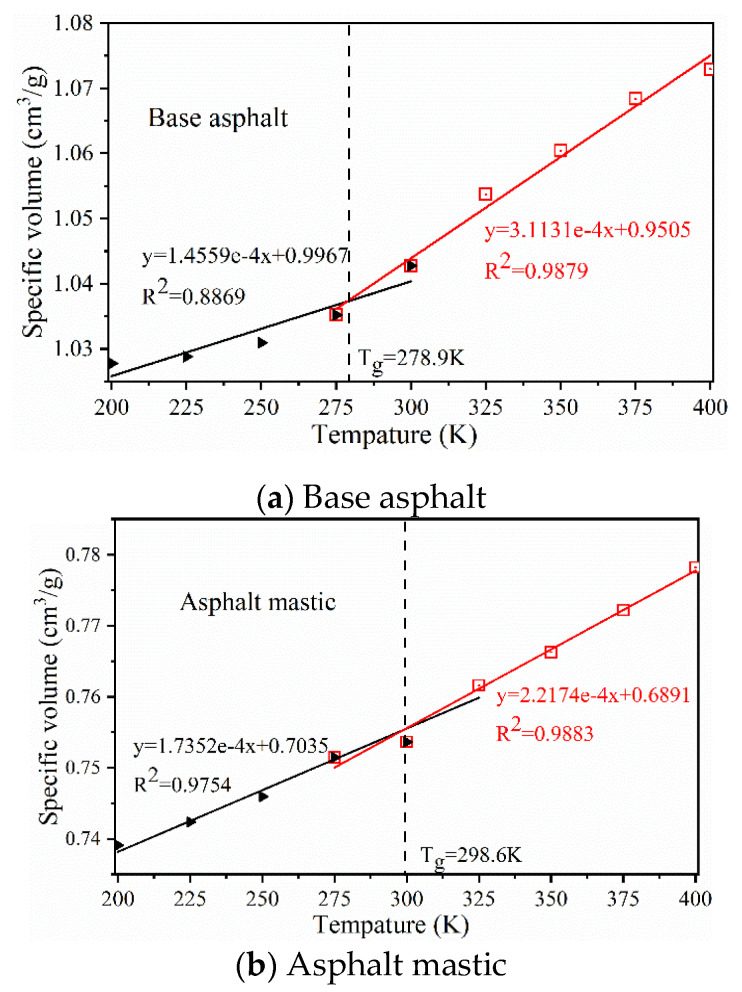
Glass-transition temperature results for base asphalt and asphalt mastic. (**a**) Base asphalt; (**b**) asphalt mastic.

The glass-transition temperature (*T_g_*) can reflect the viscoelastic properties of asphalt materials. *T_g_* is defined as the critical temperature at which the free volume inside a molecule is reduced to the point at which it can no longer accommodate the free movement of the molecular segment, leading to the molecular chain being in a frozen state. In MD simulations, *T_g_* is determined by the intersection of two asymptotes of the specific volume–temperature curve [64]. As illustrated in Figure 4, as the temperature drops from 400 K to 200 K, the specific volumes of base asphalt and asphalt mastic decrease, which indicates that the mobility of molecular chains decreases in the model systems and the phase state will be changed from a viscoelastic state into a glassy state. The *T_g_* values of base asphalt and asphalt mastic are 278.9 K and 298.6 K, respectively. The calculated *T_g_* value of base asphalt is consistent with the results, as presented in Table 2. The addition of silica particles can dramatically decrease the specific volume and increase the *T_g_* value of base asphalt, which means that the asphalt mastic would be converted into a glassy state at a higher temperature than base asphalt, because the silica particles would occupy the free volume fraction of base asphalt and reduce the moveable free space of molecules.

**Figure 5:**
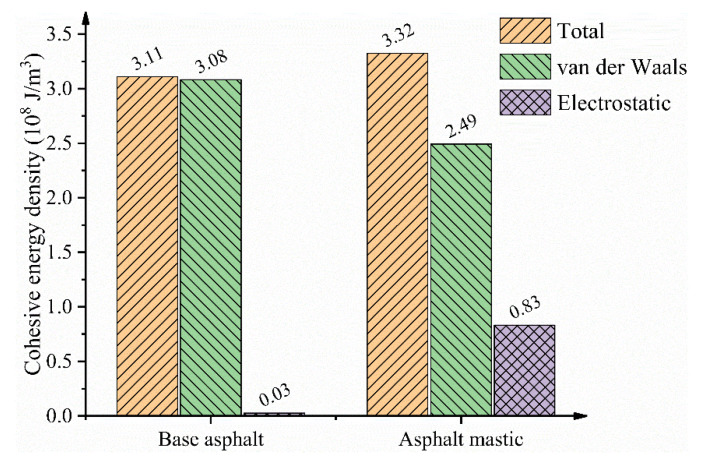
Cohesive energy density results for base asphalt and asphalt mastic.

The cohesive energy density (CED) can be applied to measure the intermolecular interaction inside the asphalt models. As shown in Figure 5, the CED value of base asphalt is 3.11 × 10^8^ J/m^3^, which is close to the results listed in Table 2. The CED value of asphalt mastic is 3.33 × 10^8^ J/m^3^, which indicates that asphalt mastic has a stronger intermolecular interaction than that of base asphalt. Meanwhile, it is observed that, when adding silica particles, the electrostatic contribution of the cohesive energy of asphalt mastic increases significantly. The main reason for this fact is that the residual charges distributed on the atoms of silica molecules would contribute to the Coulombic electrostatic interaction, even though the silica molecules are electrically neutral. In addition, the van der Waals contribution of the cohesive energy of asphalt mastic decreases to some extent, which may be attributed to the addition of silica particles that changes the molecular configuration of components in base asphalt. In conclusion, the proposed MD simulation details and the optimization process are reliable for analyzing and evaluating the thermodynamic and structural properties of asphalt models.

### 3.2. Aggregation Behaviors of Polar Components

It is well known that polar components in asphalt binders have great significance for determining the macro rheological and adhesion properties of asphalt binders. In the study, the aggregation state of polar components on the aggregate substrate was observed from the snapshots, and the radial distribution function (RDF) was analyzed for quantitively evaluating the spatial structural properties of base asphalt and asphalt mastic. The peaks on the RDF curve mean that the molecules significantly clustered each other at a certain distance, and the higher the peak value of RDF, the greater the aggregation degree. The RDF could be calculated as follows:

(1) $$g(r)=\frac{1}{\rho 4\pi r^{2}\delta r}\times \frac{\sum_{t=1}^{T}\sum_{j=1}^{N}\Delta N(r\to r+\delta r)}{N\times T},$$

where *N* is the total number of molecules; *T* is the total calculation time (ps); *r* is the radius from the target particle; δr is the designed difference in the distance; ΔN is the number of the molecules within the interval of r→r+δr; and ρ is the density of the system.

**Figure 6:**
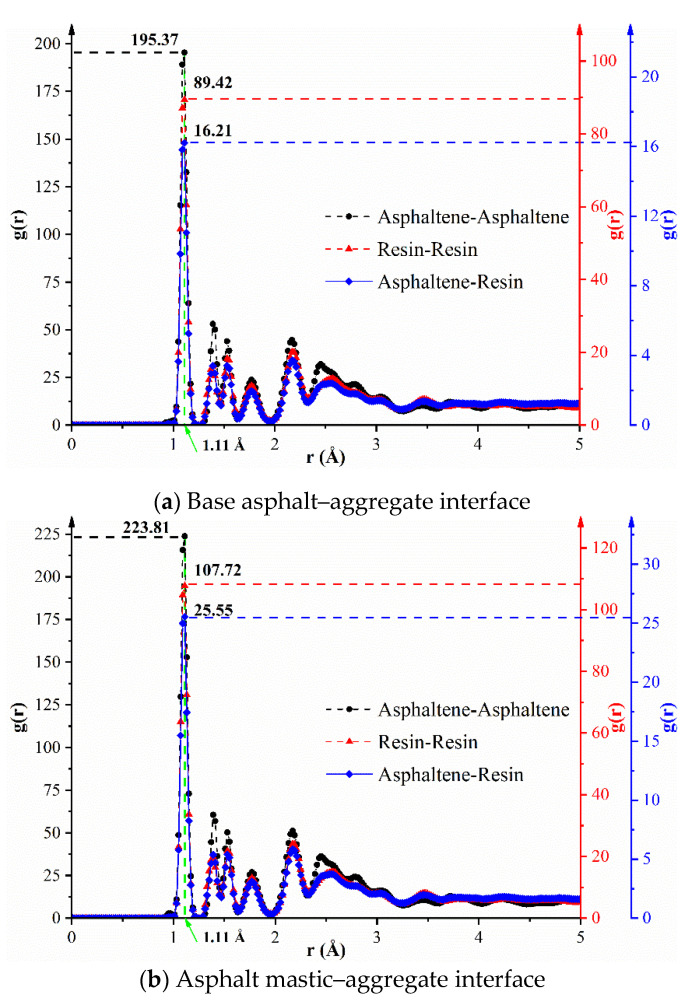
Radial distribution function (RDF) curves of asphaltene and resin. (**a**) Base asphalt–aggregate interface; (**b**) asphalt mastic–aggregate interface.

**Figure 7:**
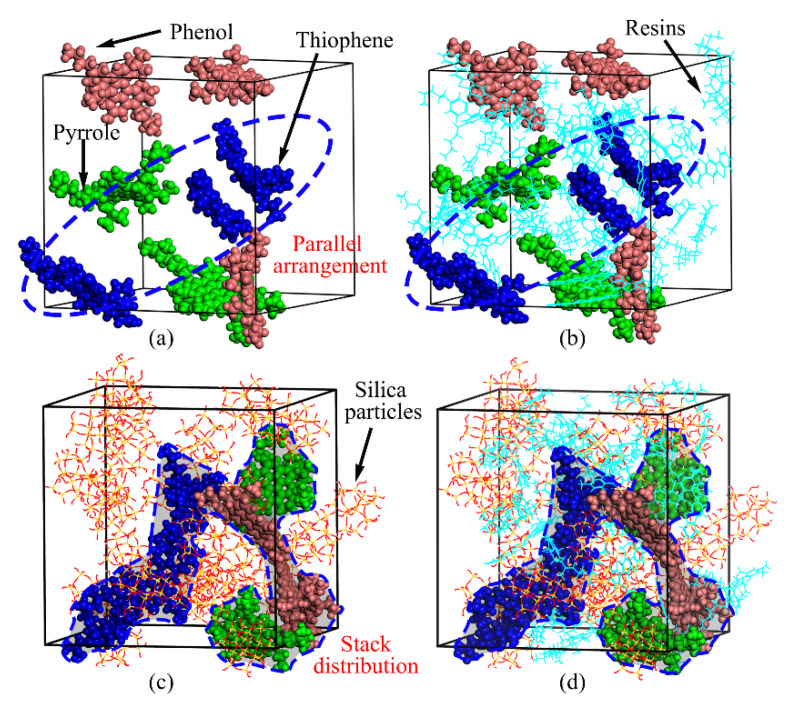
Aggregation state of polar components. (**a**) Asphaltenes without silica particles; (**b**) asphaltenes and resins without silica particles; (**c**) asphaltenes with silica particles; (**d**) asphaltenes and resins with silica particles.

The RDF curves and snapshots of asphaltene and resin before and after adding silica particles are illustrated in Figure 6 and Figure 7. As can be seen from Figure 6a, the RDF value of the asphaltene–asphaltene pair is larger than that of the resin–resin pair at the distance of 1.11 Å. Besides, the RDF values of the asphaltene–asphaltene pair and resin–resin pair are more abundant than that of the asphaltene–resin pair, which indicates that the same polar components are more likely to self-aggregate and the aggregation degree among different polar components is not distinct. Figure 6b shows the RDF curves of polar components on the aggregate substrate with the addition of silica particles, and it is found that the peak values of different pairs at 1.11 Å in asphalt mastic are higher than those in base asphalt. Accordingly, the self-aggregation behaviors of polar components could be significantly enhanced owing to the high polarity of silica particles.

The aggregation behaviors of asphaltenes and resins were observed from the snapshots of base asphalt and asphalt mastic, as illustrated in Figure 7. In Figure 7a, the asphaltenes including phenol, pyrrole, and thiophene are uniformly distributed in base asphalt, in which the thiophene molecules exhibit the most parallel distribution state. With the addition of silica particles, the asphaltenes aggregate each other to form a stack distribution around silica particles, as shown in Figure 7c. By analysis, it is believed that the effect of silica particles on adsorption of polar components, especially for asphaltenes, improves the RDF value of the asphaltene–asphaltene pair, thus leading to aggregation of the asphaltenes. Meanwhile, it can be seen from Figure 7b,d that the asphaltene molecules are uniformly dispersed within resin molecules, which is consistent with the colloidal structure theory of asphalt binder [69]. The colloidal structure of asphalt mastic has not been changed with the addition of silica particles. Moreover, it is observed that silica particles could promote the combination of asphaltene and resin, which causes them to present a more compact state and form an encapsulating region of resins around the asphaltenes, improving the stability of colloidal subcores composed of asphaltenes and resins.

### 3.3. Distribution Characteristics of Polar Components

#### 3.3.1. Relative Concentration

**Figure 8:**
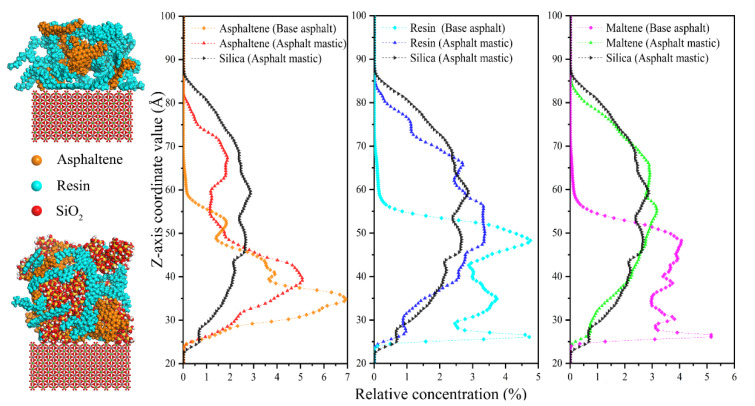
Relative concentration curves of asphalt components on the aggregate surface.

Compared with RDF, the relative concentration (RC) of asphalt components on the aggregate substrate in different directions (x, y, and z directions) could describe the distribution characteristics affected by the coupling effect of silica particles and aggregate substrate and further reflect the interfacial adhesion mechanism. In the study, the concentration profiles of asphaltene, resin, and maltene in Z directions were calculated based on the dynamic trajectory. As presented in Figure 8, as far as base asphalt is concerned, both asphaltene and resin mainly distribute at a range from 23 Å to 65 Å relative to the aggregate substrate. The distribution of resin is more uniform than asphaltene, which exhibits a characteristic of covering the distribution range of asphaltene. A “narrow and high” peak of asphaltene appears at 35 Å away from the aggregate substrate with a RC of 6.9%. The distribution of resin presents a wide state and the highest peak of RC is 4.9%. Besides, the distribution of maltene composed of resin, saturate, and aromatic is similar to that of resin. As for asphalt mastic, the distribution profile of silica particles exhibits a smooth and flat state, which indicates that silica particles are evenly dispersed in asphalt mastic. The distribution curves of polar components are close to that of silica particles to some extent, demonstrating that polar components gather around silica particles. It is worth mentioning that the addition of silica particles reduces the peak value of RC of asphaltene from 6.9% to 4.9% and shifts the corresponding peak point from 35 Å to 40 Å. The same change pattern is also observed for resin. The peak value drops from 4.9% to 3.5% as the distance of the peak point relative to the aggregate substrate increases from 25 Å to 48 Å. It is believed that the addition of silica particles significantly changes the distribution of polar components of base asphalt because of the adsorption effect of silica particles for polar components, as mentioned in the [39].

#### 3.3.2. Mean Square Displacement

The mean square displacement (MSD) was applied for characterizing the molecular mobility of asphalt components on the aggregate substrate over time. Clearly, the larger slope of the MSD curve indicates greater mobility of molecules and the MSD could be defined as Equation (2).

(2) $$MSD=\langle {\left(r_{i}(t)-r_{i}(0)\right)}^{2}\rangle ,$$

where $r_{i}(t)$ is the position vector of particle *i* at time t; $r_{i}(0)$ is the position vector of particle *i* at the initial time; and 〈〉 indicates that the squared magnitude of this vector is averaged over many such time intervals.

**Figure 9:**
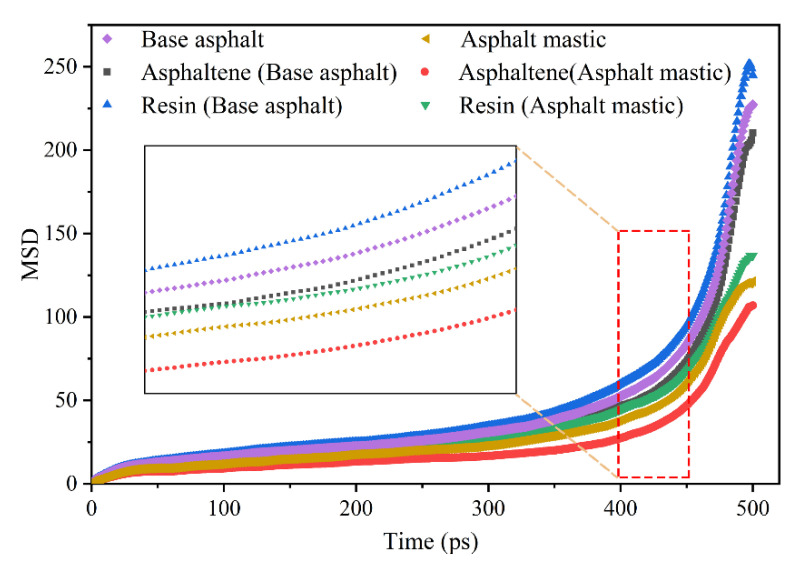
Mean square displacement (MSD) of polar components on the aggregate substrate.

**Figure 10:**
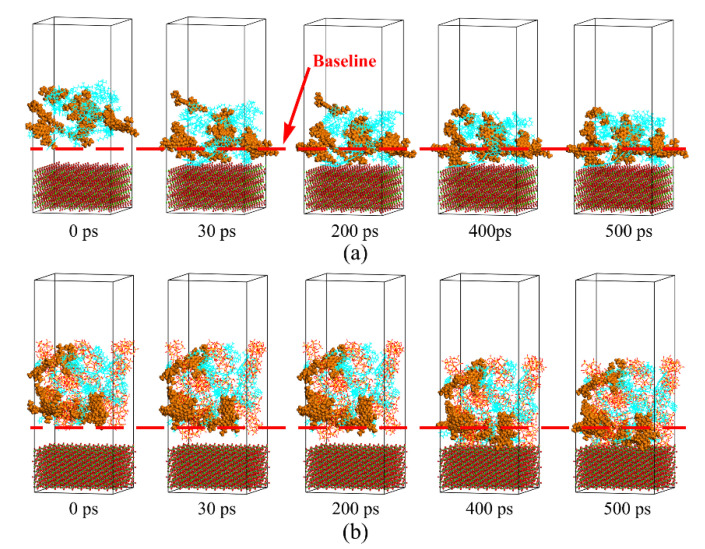
Diffusion process of polar components on the aggregate substrate. (**a**) Base asphalt–aggregate interface; (**b**) asphalt mastic–aggregate interface.

As shown in Figure 9, the resin shows greater mobility than asphaltene regardless of the presence of silica particles, because the molecular weight of resin is less than that of asphaltene. After adding silica particles, the molecular mobility of asphaltene and resin significantly declines. The reason for this phenomenon might be that the addition of silica particles increases the viscosity of base asphalt and prevents the polar components from diffusing into the aggregate substrate. As mentioned in the literature, the diffusion of asphalt binder is a process of transferring asphalt components from a rich concentration to an insufficient concentration until an equilibrium state is achieved [27]. The diffusion processes of base asphalt and asphalt mastic during simulation time are illustrated in Figure 10. The results show that the diffusion process of asphaltene, resin, and silica particles presents the same characteristic of approaching the aggregate substrate as the simulation time progresses. The polar components in base asphalt and asphalt mastic exhibit different mobility velocities during the diffusion process. As for base asphalt, asphaltene and resin rapidly approach the aggregate substrate within 30 ps and gradually stabilize after 200 ps. Meanwhile, as for asphalt mastic, the polar components and silica particles start to migrate and diffuse at 200 ps, after which the polar components gradually reach the initial equilibrium state at 400 ps. Therefore, the base asphalt exhibits a better mobility capability than the asphalt mastic from the diffusion process, which is consistent with the research results of MSD.

### 3.4. Interfacial Bonding Strength

**Figure 11:**
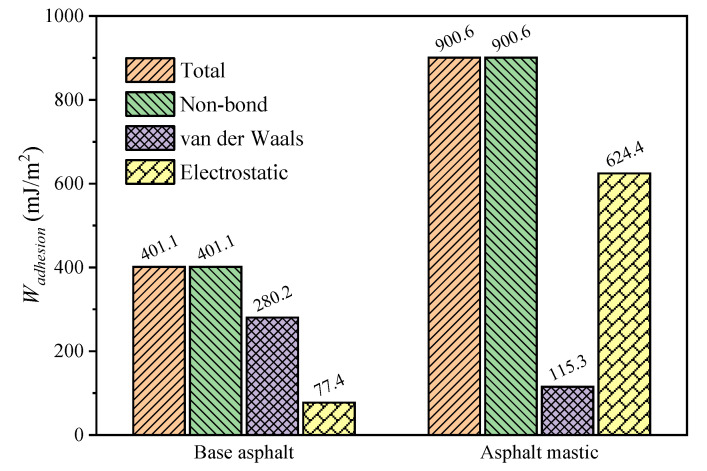
Work of adhesion of the base asphalt–aggregate interface and asphalt mastic–aggregate interface.

In order to investigate the interfacial bonding strength between asphalt and aggregate, the work of adhesion of the asphalt–aggregate interface was calculated, which is defined as the energy required to separate an interface into two free surfaces in a vacuum [70]. The work of adhesion at dry and wet conditions could be described as Equations (3) and (4), respectively. The results of the work of adhesion of the asphalt–aggregate interface are shown in Figure 11.

(3) $$W_{adhesion\_dry}=\frac{\Delta E_{inter\_ag}}{A}=\frac{E_{asphalt}+E_{aggregate}-E_{total}}{A},$$

(4) $$W_{adhesion\_wet}=\frac{\Delta E_{inter\_aw}+\Delta E_{inter\_gw}-\Delta E_{inter\_ag}}{A},$$

where $W_{adhesion\_dry}$ is the work of adhesion between asphalt and aggregate at dry condition; $\Delta E_{inter\_ag}$ is the interaction energy between asphalt and aggregate; $E_{asphalt}$ and $E_{aggregate}$ are the potential energy of individual asphalt and aggregate model, respectively; $E_{total}$ is the total potential energy of the asphalt–aggregate interface model; A is the contact area of the interface between asphalt and aggregate; $W_{adhesion\_wet}$ is the work of adhesion between asphalt and aggregate under wet conditions; $\Delta E_{inter\_aw}$ is the interaction energy between asphalt and water without aggregate; $\Delta E_{inter\_gw}$ is the interaction energy between aggregate and water without asphalt; and $\Delta E_{inter\_ag}$ is the interaction energy between asphalt and aggregate without water.

As illustrated in Figure 11, the total interfacial adhesion work is mainly composed of non-bond energy, which means that the interaction between asphalt and aggregate is mainly physical adsorption [34]. When adding silica particles, the work of adhesion of 900.6 mJ/m^2^ for the asphalt mastic–aggregate interface is more than that of 401.1 mJ/m^2^ for the base asphalt–aggregate interface. Meanwhile, it is observed that the van der Waals mainly contributes to the work of adhesion of the base asphalt–aggregate interface, and the electrostatic mainly provides the work of adhesion of the asphalt mastic–aggregate interface. The main reason for this fact is attributed to the different arrangement of polar components between base asphalt and asphalt mastic, i.e., parallel arrangement and stack distribution, as presented in Figure 7. Previous studies indicated that the “parallel aligned” and “close enough” molecules structure could provide a strong intermolecular interaction [71,72], contributing to a high van der Waals energy. Consequently, the base asphalt–aggregate interface has a higher van der Waals energy compared with the asphalt mastic–aggregate interface. On the other hand, because silica particles carry amounts of residual charges, the electrostatic energy for the asphalt mastic–aggregate interface is significantly increased with the addition of silica particles.

**Figure 12:**
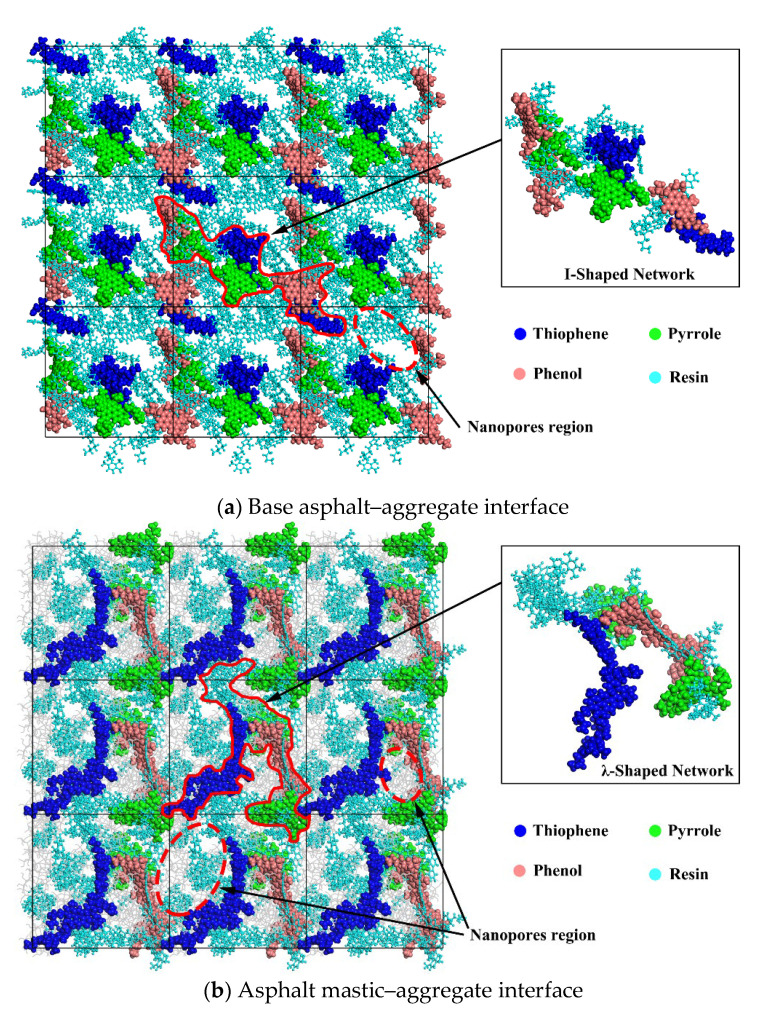
Nanostructure of the asphalt–aggregate interface. (**a**) Base asphalt–aggregate interface; (**b**) asphalt mastic–aggregate interface.

To further understand the difference of work of adhesion between the base asphalt–aggregate interface and asphalt mastic–aggregate interface, the snapshots of periodical lattices and the interfacial nanostructure composed of polar components are presented in Figure 12. As can be seen from Figure 12a, asphaltenes are interlocked with each other and surrounded by resins, forming an I-shaped network skeleton nanostructure in the base asphalt–aggregate interface. As shown in Figure 12b, the asphaltenes and resins form a λ-shaped network skeleton nanostructure in the asphalt mastic–aggregate interface owing to the adsorption of silica particles for polar components. As mentioned in [29], the nanostructures composed of polar components including asphaltenes and resins could provide van der Waals energies for interfacial adhesion. In addition, it is observed that the molecular micelles generated in adjacent periodical lattices are assembled to form a bulgy boundary, leading to the appearance of nanopores regions at the center of the bulgy boundary. As far as asphalt mastic is concerned, silica particles uniformly dispersed in asphalt mastic would inevitably fill the nanopores regions in the asphalt mastic–aggregate interface, thus providing more electrostatic energies than the base asphalt–aggregate interface, which results in the improvement of the bonding strength between asphalt mastic and aggregate.

### 3.5. Impact of Temperature and Moisture on Adhesion Behaviors

**Figure 13:**
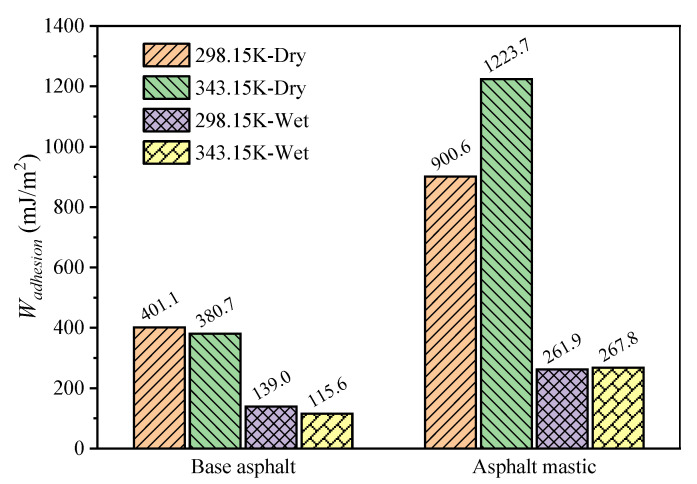
Total work of adhesion of interface models under different moisture and temperature conditions.

To investigate the effect of temperature and moisture on the adhesion behaviors of the base asphalt–aggregate interface and asphalt mastic–aggregate interface, the work of adhesion with and without moisture at 298.15 K and 343.15 K were calculated based on Equations (3) and (4), respectively. The results of the total work of adhesion are illustrated in Figure 13. Notably, the inclusion of moisture has a negative effect on the work of adhesion for the asphalt–aggregate interface, regardless of temperature conditions. For example, the work of adhesion decreases from 401.1 mJ/m^2^ to 139.0 mJ/m^2^ for the base asphalt–aggregate interface and drops from 900.6 mJ/m^2^ to 261.9 mJ/m^2^ for the asphalt mastic–aggregate interface in the presence of moisture at 298.15 K. Similar results could be found at a high temperature of 343.15 K. In particular, the loss of adhesion of the asphalt mastic–aggregate interface is more significant than that of the base asphalt–aggregate interface at 343.15 K when concerning the moisture effect. However, the condition of temperature exhibits a different impact on the work of adhesion for the base asphalt–aggregate interface and asphalt mastic–aggregate interface. Under dry conditions, the work of adhesion for the base asphalt–aggregate interface slightly decreases from 401.1 mJ/m^2^ to 380.7 mJ/m^2^ when the temperature is elevated from 298.15 K to 343.15 K, while the work of adhesion of the asphalt mastic–aggregate interface has an increase of about one-third from 900.6 mJ/m^2^ to 1223.7 mJ/m^2^. Similar phenomena of base asphalt–aggregate interface and asphalt mastic–aggregate interface are observed under wet conditions. The results indicate that the increase of temperature has a positive effect on asphalt mastic–aggregate interfacial adhesion, but a negative effect on that of the base asphalt–aggregate interface.

**Figure 14:**
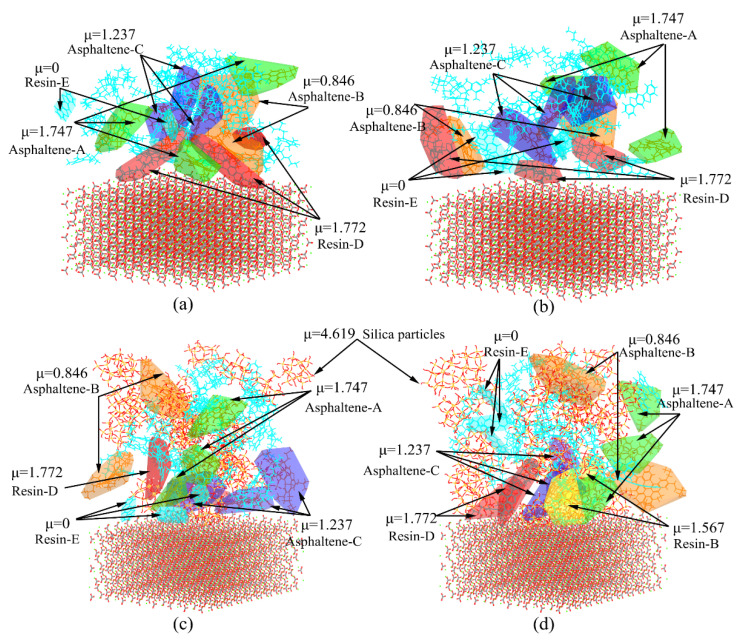
Distribution of polar components in the asphalt–aggregate interface at different temperatures. (**a**) Base asphalt–aggregate interface at 298.15 K; (**b**) base asphalt–aggregate interface at 343.15 K; (**c**) asphalt mastic–aggregate interface at 298.15 K; (**d**) asphalt mastic–aggregate interface at 343.15 K.

A recent study indicated that the molecular orientation theory could effectively explain the adhesion behaviors of the asphalt–aggregate interface by means of dipole moment. The polar molecules with a higher dipole moment undergo an orientated adsorption at the proximal end of the aggregate surface, contributing to the bonding strength between the asphalt and aggregate [27]. The dipole moments of asphalt components and silica particles are listed in Table 3. The asphalt–aggregate interface marked with a dipole moment at temperatures of 298.15 K and 343.15 K is illustrated in Figure 14. As shown in Figure 14a, the polar components with a higher dipole moment are relatively aggregated and adsorbed near the proximal end of the aggregate substrate at the temperature of 298.15 K. With the increase of temperature, the polar components tend to disperse and keep away from the aggregate substrate at the temperature of 343.15 K, as illustrated in Figure 14b. The main reason for this fact is that the asphaltenes are quite sensitive to the temperature, which would prompt asphaltenes to separate to a certain distance and present a dispersion state. The results are consistent with the finding from the previous study [73]. The molecular arrangement of polar components changes with the increase of temperature. For example, the Resin-E molecules with zero dipole moment are much closer to the aggregate substrate at the temperature of 343.15 K than at the temperature of 298.15 K. The above results would lead to a weaker orientated adsorption of polar components for the aggregate substrate and reduce the work of adhesion of the base asphalt–aggregate interface at the critical high temperature of an in-service pavement.

It is worth mentioning that the characteristics occurring in the base asphalt–aggregate interface could be altered with the addition of silica particles. As can be seen from Figure 14c,d, with the increase of temperature from 298.15 K to 343.15 K, the polar components with a low dipole moment stay away from the aggregate substrate, while polar components with a high dipole moment are more prone to be close to the aggregate substrate. As an illustration, the Resin-D and Resin-B molecules are closer to the aggregate substrate and the Resin-E molecules with zero dipole moment are away from the proximal end of the aggregate surface at a high temperature of 343.15 K. Consequently, the asphalt mastic–aggregate interface at the high temperature would exhibit the superior work of adhesion. Additionally, silica particles with a relatively higher dipole moment value of 4.619 debye in asphalt mastic would provide a stronger orientated adsorption for the aggregate substrate, which contributes to the improvement of work of adhesion for the asphalt mastic–aggregate interface.

**Figure 15:**
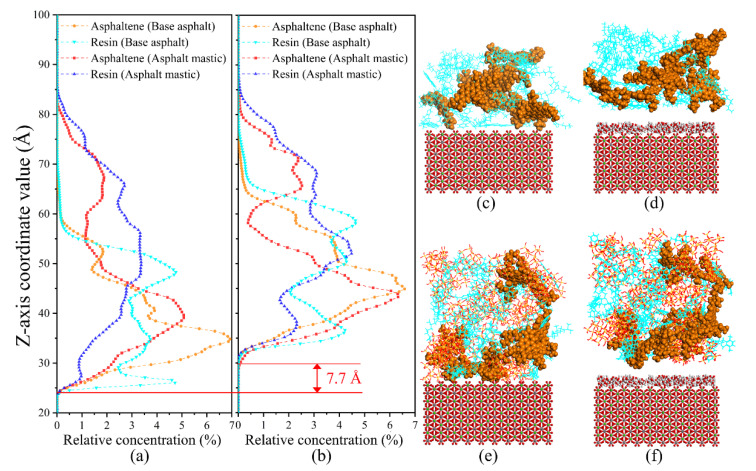
The relative concentration (RC) curves and snapshots of the asphalt–aggregate interface under dry and wet conditions. (**a**) RC curves under dry condition; (**b**) RC curves under wet condition; (**c**) base asphalt–aggregate interface under dry condition; (**d**) base asphalt–aggregate interface under wet condition; (**e**) asphalt mastic–aggregate interface under dry condition; (**f**) asphalt mastic–aggregate interface under wet condition.

The reason for the fact that the moisture sharply decreases the work of adhesion for the asphalt–aggregate interface could be explained in Figure 15. It is observed that the presence of moisture increases the initial distance of the appearance of polar components on the aggregate substrate by 7.7 Å, as shown in Figure 15a,b. According to the molecular orientation theory, an interface with a larger gap would result in less orientated adsorption between polar components and aggregate substrate, reducing the work of adhesion for the asphalt–aggregate interface. Meanwhile, because of the hydrophobicity of the asphalt and the hydrophilicity of the aggregate [67], moisture has an intrinsic ability to separate asphalt from aggregate surface, as shown in Figure 15c–f. In conclusion, the work of adhesion of the asphalt–aggregate interface sharply decreases with the presence of moisture.

**Figure 16:**
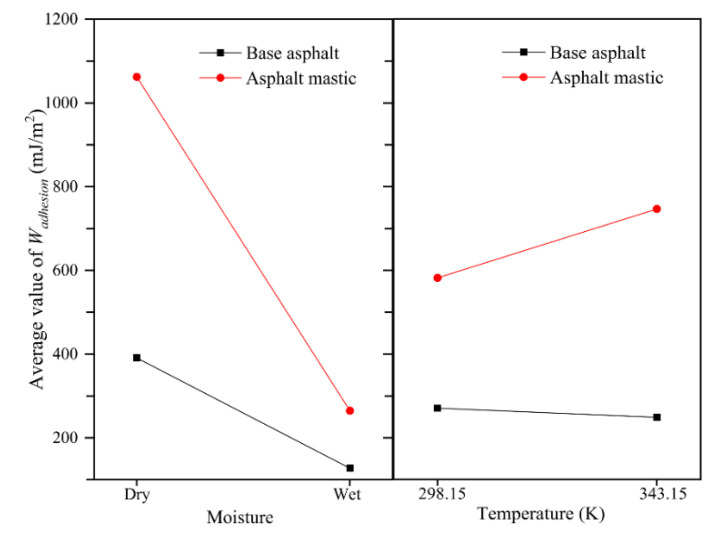
Coupling effect of moisture and temperature on interfacial adhesion behaviors.

To further explore the coupling effect of moisture and temperature on the adhesion behaviors of the asphalt–aggregate interface, the average values of work of adhesion for the asphalt–aggregate interface at moisture and temperature conditions were calculated, respectively, as illustrated in Figure 16. For example, when investigating the effect of moisture, the work of adhesion of the base asphalt–aggregate interface was the average value of the calculated values at 298.15 K and 343.15 K.

As shown in Figure 16, as for base asphalt–aggregate interface, both moisture and high temperature exhibit negative effects on the work of adhesion. The weakening effect of moisture on adhesion is more significant than that of temperature as the curve of work of adhesion considering the moisture condition exhibits a more distinct decrease slope than that considering the temperature condition. Nonetheless, the presence of moisture deteriorates the adhesion of the asphalt mastic–aggregate interface, while the increase of temperature enhances the interfacial adhesion to some extent. The downward slope for moisture is more remarkable than the upward slope for temperature, demonstrating that the moisture is the crucial factor to deteriorate the adhesion between asphalt mastic and aggregate. With further observation, asphalt mastic is more sensitive to interfacial moisture damage than base asphalt, as the decline slope of the asphalt mastic–aggregate interface is steeper than that of the base asphalt–aggregate interface. The fact would provide an essential basis for understanding the severe moisture damage issues of asphalt mixtures. It is asphalt mastic rather than asphalt binder that plays the role of bonding aggregates and filling voids in asphalt mixtures. The traditional adhesion tests of asphalt materials usually focused on the bonding strength between asphalt binders and aggregates, which would overestimate the moisture damage resistance of asphalt mixtures and could not accurately reflect the prone early moisture distress occurring in asphalt pavement. Consequently, the asphalt mastic–aggregate interface model developed in this study is reasonable and reliable to understand the actual adhesion and debonding behaviors of asphalt mixtures during the service life.

## 4. Conclusions

In this study, the adhesion behaviors of the asphalt mastic–aggregate interface were comprehensively explored by MD simulations from the perspective of the molecular arrangement characteristics and interfacial nanostructures of the asphalt mastic–aggregate interface. Based on the simulated results, the main conclusions are summarized as follows:

1. The asphalt mastic model under the actual F/A condition is established and validated by analyzing the difference of thermodynamic properties between asphalt mastic and base asphalt.
2. The self-aggregation behaviors of polar components are significantly enhanced with the addition of silica particles. Because of the high polarity of silica particles, the molecular arrangement of polar components is changed from “parallel arrangement” into “stack distribution”, which results in the decrease of van der Waals energy.
3. Silica particles could significantly change the distribution of polar components of base asphalt because of the adsorption effect of silica particles, and polar components adsorbed around the evenly dispersed silica particles in asphalt mastic exhibit a more uniform distribution state and lower mobility capability than base asphalt.
4. The addition of silica particles with amounts of residual charges dramatically increases the electrostatic energy of the asphalt mastic–aggregate interface, thus strengthening the adhesion between asphalt mastic and aggregate.
5. The coupling effect of moisture and temperature indicates that moisture could dramatically deteriorate the adhesion between asphalt and aggregate, especially for the asphalt mastic–aggregate interface. The increase of temperature enhances the work of adhesion of the asphalt mastic–aggregate interface, which is opposite to that of the base asphalt–aggregate interface.

## Figures and Tables

**Table 1:** Molecular compositions and characteristics of the studied base asphalt.

SARA Fractions	Molecules	Molecules Number	Molecular Formula	Molar Mass (g/mol)	Mass Fraction (%)
Saturates (S)	Squalane	4	C_30_H_62_	422.9	5.2
	Hopane	4	C_35_H_62_	482.8	5.8
Aromatics (A)	PHPN	11	C_35_H_44_	464.8	15.7
	DOCHN	13	C_30_H_46_	406.8	16.2
Resins (R)	Pyridinohopane	4	C_36_H_57_N	530.9	6.2
	Thio-isorenieratane	4	C_40_H_60_S	572.9	7.0
	Trimethylbenzene-oxane	5	C_29_H_50_O	414.7	6.4
	Quinolinohopane	4	C_40_H_59_N	554.0	6.8
	Benzobisbenzothiophene	15	C_18_H_10_S_2_	290.4	13.4
Asphaltenes (A)	Phenol	3	C_42_H_54_O	575	5.3
	Pyrrole	2	C_66_H_81_N	888.5	5.5
	Thiophene	3	C_51_H_62_S	707.2	6.5

**Table 2:** Calculated results of base asphalt in molecular dynamic (MD) simulations.

Properties	Calculated Results	Simulated Results in the Literatures
Density (298.15 K)	0.998	0.92 (Long et al. [48]); 0.997 (Khabaz and Khare [65]);0.981 (Gao et al. [66])
Glass-transition temperature (K)	278.9	278.66 (Zhu et al. [41]); 275(Xu [67])
Cohesive energy density (10^8^ J/m^3^)	3.11	3.32 (Xu and Wang [32]); 3.21(Wang et al. [68])

**Table 3:** The dipole moment of asphalt components and silica particles.

Components	Number	Molecules	Molecular Formula	Dipole Moment (debye)
Saturates	A	Squalane	C_30_H_62_	0.077
	B	Hopane	C_35_H_62_	0.052
Aromatics	A	PHPN	C_35_H_44_	0.364
	B	DOCHN	C_30_H_46_	0.662
Resins	A	Pyridinohopane	C_36_H_57_N	2.123
	B	Thio-isorenieratane	C_40_H_60_S	1.567
	C	Trimethylbenzene-oxane	C_29_H_50_O	1.170
	D	Quinolinohopane	C_40_H_59_N	1.772
	E	Benzobisbenzothiophene	C_18_H_10_S_2_	0
Asphaltenes	A	Phenol	C_42_H_54_O	1.747
	B	Pyrrole	C_66_H_81_N	0.846
	C	Thiophene	C_51_H_62_S	1.237
Silica particles	/	Silicon dioxide	SiO_2_	4.619
